# Implementation of a stepped-wedge cluster randomized design in routine public health practice: design and application for a tuberculosis (TB) household contact study in a high burden area of Lima, Peru

**DOI:** 10.1186/s12889-015-1883-2

**Published:** 2015-06-26

**Authors:** Lena Shah, Marlene Rojas, Oscar Mori, Carlos Zamudio, Jay S. Kaufman, Larissa Otero, Eduardo Gotuzzo, Carlos Seas, Timothy F. Brewer

**Affiliations:** Department of Epidemiology, Biostatistics & Occupational Health, McGill University, Purvis Hall, 1020 Pine Ave. West, Montreal, QC Canada; Red de Salud de San Juan de Lurigancho, Dirección de Salud Lima IV Este, Ministerio de Salud, Lima, Peru; Instituto de Medicina Tropical Alexander von Humboldt, Universidad Peruana Cayetano Heredia, Lima, Peru; Departamento de Enfermedades Infecciosas, Tropicales y Dermatológicas, Hospital Nacional Cayetano Heredia, Lima, Peru; Department of Medicine, David Geffen School of Medicine, University of California, Los Angeles, CA USA

**Keywords:** *Stepped-wedge*, *Active case finding*, *Tuberculosis*

## Abstract

**Background:**

We designed a pragmatic stepped-wedge cluster randomized controlled trial in order to evaluate provider-initiated evaluation of household contacts (HCs) of smear positive tuberculosis (TB) cases within a routine TB program in Lima, Peru.

**Methods/Design:**

National TB program (NTP) officers of San Juan de Lurigancho District (Lima, Peru) and university-based researchers jointly designed a pragmatic stepped-wedge cluster randomized trial design in order to evaluate a planned active case finding (ACF) program for all HCs of smear-positive TB cases in 34 district healthcare centres. Randomization of time to intervention initiation was stratified by health centre TB case rate. The ACF intervention included provider-initiated home visits of all new sputum smear positive TB patients in order to evaluate household contacts for active TB. Active TB was diagnosed using symptom screening, sputum screening, chest x-ray and clinical evaluation. Once initiated, ACF was provided by NTP staff and integrated into the routine DOTS TB program activities.

**Discussion:**

This study protocol describes the pragmatic stepped-wedge cluster randomized trial of active household contact evaluations within an NTP. The stepped-wedge design met overlapping needs of local TB programmers and researchers to adequately evaluate the large-scale roll out of a new control program in a TB endemic setting. Multiple planning meetings were required to develop the necessary networks and in order to understand the operations, needs and goals of the NTP staff and researchers collaborating on this project. The advantages and challenges of using this study design in practice and within existing routine TB programs in a middle-income country context are discussed.

**Trial registration:**

ClinicalTrials.gov NCT02174380. Registered 24 Jun 2014

## Background

Randomized controlled trials (RCTs) are considered the ‘gold standard’ study design to determine the efficacy of health care interventions [1]. High quality RCTs require particularly stringent study contexts in order to optimize their internal validity. For many public health interventions, particularly those deployed in low-resource settings, efficacy findings from rigorous research contexts do not necessarily align with those observed under the conditions and contexts in which the intervention is to be operationalized [2]. Pragmatic RCTs were initially conceived to allow for the inclusion of a more realistic study environment, for example including the intervention’s study population. Nevertheless, these studies are limited in their ability to approximate the true, real-world effectiveness of a public health intervention once it is assimilated into routine public health practice or implemented on a large-scale across populations [3].

In practice, public health interventions may be rapidly deployed on the basis of weak or limited pre-existing trial data if they are believed to be of more benefit than harm [4, 5]. However, the decision to undertake a large-scale roll out of a new public health intervention requires a corresponding investment of public resources. Depending on the type of intervention, this investment could range from costs of increasing the number trained public health staff to costs of programmatic materials including diagnostic tests or disease treatments. Therefore, public health interventions should be evaluated as they are implemented and embedded into local health infrastructure in order to examine the contextual effectiveness and to appropriately support evidence-based public health decision-making [3, 6].

The stepped-wedge variant of the cluster randomized controlled trial (CRCT) is increasingly used in pragmatic studies [7–19]. In a classic parallel CRCT design commonly used in community intervention effectiveness trials, groups of individuals or clusters (e.g. health centres) are randomly assigned to either the intervention or control arm for the entire study period [20]. In the stepped-wedge CRCT design, clusters are randomly assigned to start the intervention at different times (unidirectional cross-over trial) so that by the end of the follow-up period all clusters have initiated the intervention [7, 8, 20–22]. The integration of a staggered initiation time of the intervention in the stepped-wedge design is particularly useful if withholding the intervention is not considered equitable or for when it is difficult logistically to simultaneously initiate an intervention over a large population [8, 23]. However, very few published studies using a stepped-wedge design have been undertaken in the context of infectious disease control programs or for interventions embedded within routine public health systems in low- and middle-income countries (LMICs) [24]. Amongst these, even fewer have shared information on the design, planning, implementation, strengths, challenges and practical issues encountered in undertaking the stepped-wedge design in this context [11].

In 2012, the World Health Organization (WHO) released its first comprehensive recommendations for screening all household contacts (HCs) of persons with infectious pulmonary tuberculosis (TB) in LMICs [25]. This new guideline is in response to slow declines in TB rates and the increasing concern of TB drug resistant strains spread in areas with well-established directly observed treatment strategies (DOTS). Until recently in LMICs, DOTS focus primarily on the adequate management of TB cases once they self-report for diagnosis; recommended screening was prioritized for select high-risk groups such as children < 5 years old or individuals co-infected with human immunodeficiency virus (HIV). Despite these new expanded recommendations, a substantial gap in evidence regarding the effectiveness of household contact tracing within routine LMIC TB programs remains. In recognition of this limitation, the WHO recommendations called for operational studies of household and close contact tracing activities, including through stepped-wedge designed studies, to help guide future recommendations [25]. We designed a pragmatic stepped-wedge CRCT for active case finding among HCs of index TB cases in comparison with the detection of secondary TB cases using the routine passive detection of TB cases. The strengths, challenges and practical issues of implementing the stepped-wedge design are discussed.

## Methods

### Study context

TB is caused by the pathogen *Mycobacterium tuberculosis* which is spread primarily when a smear-positive (i.e. high bacillary load in sputum samples) pulmonary TB case coughs or exhales aerosolized droplets which are then inhaled by their close contacts. Household contacts (HCs) of smear-positive TB patients are at greater risk for acquiring TB infection and disease than the general community because of their duration and proximity of contact to infectious cases [26, 27].

DOTS refers to both a TB treatment program (including the direct observation of short-course therapy for treatment) and the first component of WHO’s global STOP TB control strategy adopted by many national TB programs (NTPs) globally, including in Peru. Since the early 1990’s, Peru has run an established DOTS program that has consistently met WHO operational performance indicator targets. Despite a standardized operational DOTS program, the goal of TB elimination in Peru remains elusive. In addition to new diagnostics, therapeutics and the current passive detection of TB cases using sputum microscopy, expanding case finding activities is of particular interest where the basic core NTP program has been implemented, as is the case in Peru.

In 2013, Peru reported an average TB incidence of 99 per 100,000 population, which is amongst the highest in the Americas. Unlike the TB epidemic in Sub-Saharan Africa, HIV is not a primary driver of the epidemic in Peru (HIV rates among TB cases are <3 %) [28, 29]. In 2010, over 1,800 new cases of TB (170 per 100,000 population) were identified in San Juan de Lurigancho (SJL), Northern Lima’s most densely population and largest district (population: 900,000, area: 131.25 km^2^) [30]. In SJL, nearly 30 % of health centres reported an incidence ranging between 200 and 400 per 100,000 population [31].

The Peruvian Ministry of Health operates its NTP in SJL through 34 health care facilities (20 community health clinics (CHCs), 14 health posts) and 1 hospital [32]. The NTP DOTS program, including TB treatment, follow-up visits, management of clinical and programmatic records and counseling occur within a designated outpatient TB program office within each of these health care facilities. The routine public health system in SJL includes the self reporting of HCs of TB cases for symptom screening. In 2010, over 60 % of eligible HCs presented to district clinics for TB screening [31]. In each health centre, typically a physician, nurse and nurse’s aide are responsible for the TB program activities. In smaller health centres with fewer TB cases, the staff is responsible for multiple public health programs within the centre, while in larger centres with numerous TB cases, full-time staff are allocated to the TB programs.

### Intervention arm

**Fig. 1:**
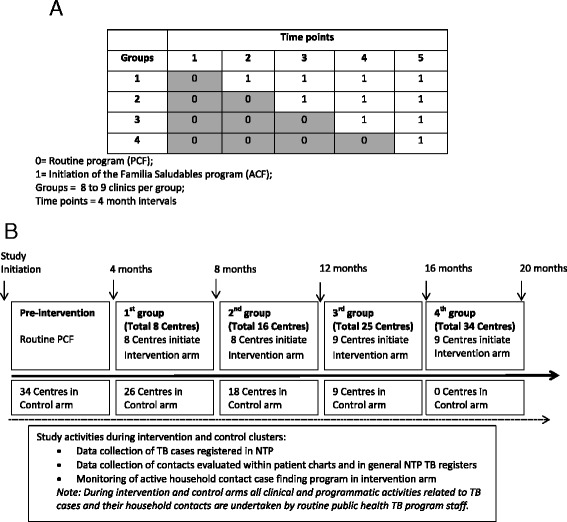
**a** Stepped-wedge implementation design. **b** Stepped-wedge implementation during study period

In the intervention arm, The SJL NTP program initiated an active case finding (ACF) program, entitled “*Familia saludables de contactos de tuberculosis*”or “Healthy Families of TB Cases”, which includes visits by a TB program nurse to households of all newly diagnosed smear-positive TB cases enrolled in DOTS treatment within an SJL NTP clinic (Fig. 1). During the home visit, NTP staff evaluates all HCs for symptoms of active TB. Any person reporting cough for over 14 days is asked to provide a spot sputum for microscopy and referred to the clinic for chest x-ray and clinical evaluation. All HCs less than 16 years old are referred to the health centre for chest x-ray, pediatric clinical evaluation and initiation of treatment for active or latent TB as required. Counseling including TB infection control practices and importance of diagnosis and treatment completion for TB cases is provided to household members (Table 1).

**Table 1:** Definitions of cases, contacts, intervention and comparator arms

Study Definitions	
TB case	A definite TB case is defined as an individual with newly diagnosed smear-positive or culture positive TB. Smear-negative individuals meeting NTP clinical guidelines for TB (cough >14 days duration with or without the presence of chest pain, fever, haemoptysis (blood in sputum), night sweats or fatigue and/or weight loss) [33, 40] were classified as probable cases.
Household Contact	Household contact is defined as living and sleeping at the same dwelling/property as the respective index case at the time of diagnosis, sharing kitchen and bathroom facilities [28, 33, 41].
Routine practice comparator	Passive case finding is the current NTP DOTS program of symptomatic persons voluntarily self-reporting to the health system for diagnosis of TB and initiation of chemotherapy [42]. Newly diagnosed and retreated smear positive TB cases enrolled in DOTS treatment at SJL NTP clinics are asked to name their HCs and encouraged to tell household members ≥15 years with cough >14 days to self-report to the clinic for evaluation. All children <15, with or without symptoms, are encouraged to attend clinic for evaluation for latent or active TB (as per DISA NTP guidelines). TB evaluation at clinics includes sputum smear microscopy, chest x-ray and clinical evaluation.
Intervention – active case finding of HCs – *Familia Saludables de contactos de TB* Program (Fig. 2)	The DISA NTP proposes the *Familia Saludables de contactos de TB* program which includes households visits of all newly diagnosed TB cases enrolled in DOTS treatment within a DISA NTP clinic. During the home visit NTP staff evaluates all HCs for symptoms of active TB. Any person reporting cough for >14 days are asked to provide a spot sputum for microscopy and referred to the clinic for chest x-ray and clinical evaluation. All HCs ≤15 years are referred to the health centre for chest x-ray, pediatric clinical evaluation and initiation of treatment for active or latent TB as required. Counseling including TB infection control practices and importance of diagnosis and treatment completion for TB cases is provided to household members. The ACF home visit is to be repeated at three times, within a month of the time the index TB case initiates treatment, at 3 months and at 6 months.

### Control arm

In the control arm (pre-intervention basic TB program), the routine DOTS program includes detection of TB cases through the self presentation of symptomatic persons, including HCs, for evaluation for TB at a NTP clinic. This approach is standard for TB control programs in most LMICs with high rates of TB. Previously in SJL, when household visits were used for TB related activities, they were primarily used to verify the jurisdiction of residence of the case and to capture any TB patients who were missing doses of their TB treatment.

### Stepped wedge design

**Fig. 2:**
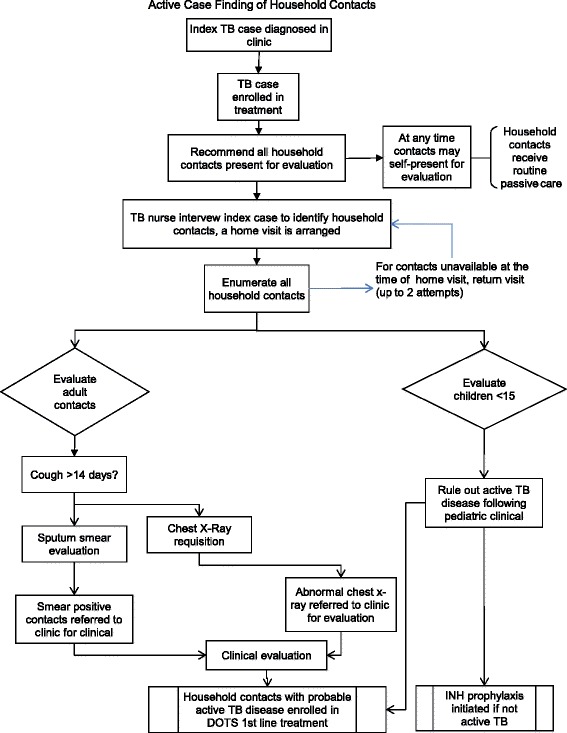
Diagram schematic of the active case finding program implemented in SJL district

Following a baseline pre-intervention data collection period, the 34 healthcare centres (excluding the hospital) were randomized to initiate the ACF program in groups of 8 or 9 clinics at four-month intervals (Fig. 2). While waiting to roll-over to the ACF home visitation intervention program, clinics continued with the routine DOTS standard of care program. A total of 20 months was required, including the four month baseline pre-roll out period, plus each of the four cross-over time points, until the ACF program was implemented and integrated into the TB programs in all SJL health centres.

### Participants

Index TB cases in SJL are included if they are greater than16 years old, sputum smear-positive and report having at least one HC upon questioning. All TB HCs identified are included if they meet the program HC definition and are not currently under TB treatment [33]. Patients diagnosed outside of the Ministry of Health NTP, such as those imprisoned, institutionalized or attending private clinics, are excluded unless referred and registered into the Ministry of Health NTP DOTS program.

### Randomization and stratification

Randomization of health centres was stratified by TB incidence rate tertiles to achieve a balance of TB burden in clusters crossing over to the intervention arm at each time period (step). Clinics are categorized into one of following TB rate strata: less than 100/100,000, between 100 and 200/100,000 and greater than 200/100,000 population. Health centres within strata were randomized to the time of intervention initiation using a random number generated sequence in R Software [34].

### Blinding

Study investigators, DISA NTP TB program staff, TB cases and their HCs were not blinded to the initiation of the intervention. Clinics were notified one month prior to their crossover date to initiate program planning. To minimize potential ascertainment bias, TB disease was determined by laboratory diagnosis [35]. While the intervention and control arms were integrated into routine public health practice, there was potential for behaviour differences given our supervision of health centers, simply from staff knowing their work is monitored. Data collection in all clinics was initiated several months prior to the initial rollout of the intervention to reduce biases incurred from study research team observing practices during the intervention period.

### Prevention of contamination

Health centre TB program staff are trained to prospectively enroll newly diagnosed TB cases beginning on the first of the month they initiate the intervention. During crossover, there was a risk of contamination from the time the NTP staff were trained to implement the intervention prior to the clinic crossover date to the new program. Training and monitoring were scheduled as closely as possible to the intervention start date to minimize this effect (within two weeks). Contamination between clinics was considered to be minimal, as cases and contacts are required to initiate TB treatment within the clinic catchment area of their primary residence, primarily for administrative reasons. TB cases diagnosed in other clinics within the district or in other districts are referred to the designated clinic of their primary residence prior to initiation of NTP provided TB treatment or shortly thereafter.

### TB Diagnosis

Throughout the study, both cases and contacts were diagnosed using existing local NTP diagnostic practices. These routine practices include symptom screening, sputum smear microscopy, radiography, clinical evaluation, culture-confirmation and drug susceptibility testing. Though laboratory based rapid TB diagnostic methods are in the process of being integrated into Peru’s NTP, tests such as GenXpert MTB/RIF and point-of-care rapid diagnostics for TB are not in use in SJL.

### Power analysis

Accounting for the stepped-wedge design and the varying cluster sizes within the sample, with a fixed number of clusters (n = 34 health centres) and a power of 90 %, the study is powered to detect a 3 to 4 % difference in the proportion of HCs detected with TB between the intervention (ACF) and control arms. Based on previous studies, this is sufficient power to determine an effect that would be of clinical and programmatic importance [36, 37]. The primary outcome is the rate of TB among HCs of TB patients, yields in % positive for TB by total number of contacts evaluated, and the secondary outcome is the number of contact tested per index case tested.

### Consent statement

Verbal consent was solicited from index TB cases and contacts, including for household visits by the TB program staff and for clinical evaluations as part of the Ministry of Health routine public health program. Ethical approvals were granted by the Human Ethics Research Boards of the McGill University Health Centre (Montreal, Quebec, Canada), Universidad Peruana Cayetano Heredia (Lima, Peru) and Dirección de Salud Lima IV Este (Lima, Peru).

### Trial status

At the time of this publication, the pragmatic stepped-wedge CRCT is underway across SJL health centers, Lima, Peru with funds from the Canadian Institutes of Health Research (CIHR). The study is registered in the Clinical Trials.gov database (NCT02174380). Implementation and data collection have initiated. Study outcomes, data cleaning and analysis are pending.

## Discussion

This pragmatic stepped-wedge CRCT protocol is among the very few undertaken in an operational public health program in a LMIC [38]. The following discussion highlights the planning stages and development of this collaborative study protocol, the initiation and implementation of this study, including the strengths and challenges of using the stepped-wedge design in the implementation of an intervention within a public health program under real-world conditions (Table 2).

**Table 2:** Key advantages and challenges related to public health implementation in a stepped-wedge pragmatic CRCT

Study Feature	Advantages	Challenges	Implications in current protocol
Stepped or staggered implementation	• Allows for the incremental introduction of the intervention or program.	• Multiple training and initiation must be undertaken at each step (or cluster crossover).	• The phased initiation is crucial to undertaking implementation across all 34 health centres all of which require individual training and monitoring which would not have been feasible in a full roll out or even a parallel CRCT.
		• Multiple measurements is resource intensive throughout the entire study period during crossover or initiation and throughout all steps.	
	• Flexible design that can be modified for amount of steps and clusters based on need or manageability.		
		• Higher complexity in biostatistical analysis	
	• Higher quality evidence than observational and non-randomized pretest-posttest designs.		
	• All centers are intervention and controls.		
Implementation in routine conditions	• The stepped-wedge study design allows for all partners to reach their specific goals and obtain the evidence they need from their perspective.	• Routine public health in LMICs is subject to many fiscal, political and programmatic pressures, however methodologic rigour is still required in this design and should be adhered to at least during the study period.	• The stepped-wedge pragmatic RCT is the most suited so all partners could benefit from high quality evidence, yet pragmatic utility.
	• The design can be used to provide real world evidence of effectiveness by using a staggered implementation when a full population based intervention is planned. This allows for evidence that may not exist otherwise.		• Researchers had identified the major gaps in available high quality research evidence for the routine systematic evaluation of HCs of TB cases within the context of resource constrained TB endemic areas. In parallel, SJL district local NTP programmers had identified the urgent need to implement a program to actively evaluate HCs of TB cases undergoing treatment; there was a local programmatic concern of cases that were linked through familial or household contact, and the underperformance of their current passive approach for achieving screening of HCs.
		• Researchers desire randomization, unbiased allocation of health centers.	
		• Data quality needs for research design may be above daily programmatic requirements (data quality control).	
Overall Design	• Easy to integrate design, a modification of existing RCT and CRCT	• Methodological complexities to power and analyses of stepped-wedge designs	• Many of the authors of stepped-wedge design papers have contributed to the use and the methods for this design. On the other hand every study has its individual specific needs and we required specialized guidance in terms of the overall framework of the roll out, the frequency and duration of steps.
		• Fewer experts with technical and practical experience using stepped-wedge designs	
	• RCT experts and methodologists widely available		
			• Because of its relatively recent use, published literature including many of the published stepped-wedge trial protocols in peer reviewed literature were relied upon.
		• Unlike traditional individual randomized controlled trials and cluster randomized controlled trials, less is formally taught in research training and in operational training on the design and requirements for methodological rigour.	
		• The nomenclature is complex and could be confusing to stakeholder. Several terms have been used for the same design modification, including implementation trial, randomized start or staggered start trial, delayed designs, step or stepped-wedge designs amongst others [21, 22]	
Longer trial length	• Focuses on a smaller subset of the intervention groups at time	• Population based intervention implementation requires programmatic and research supervision, throughout the duration of the entire study.	• As it is phased in, the monitoring, supervision and training can be improved and also any major problems, political, technical or resources can be identified to improve the implementation.
	• Improves adherence by allowing for intensive monitoring and supervision of groups		
		• Longer trial period is also associated with increased intensity and duration of labour	
			• There are great challenges to sustaining monitoring efforts, in practice far more training and monitoring and supervision is required then initially estimated.
	• Improves quality of training including smaller size of training groups and ability to integrate peer to peer support and hands on training in the field		
			• Research components can increase burden and the longer duration can lead to exhaustion for researchers, programmers and local health care staff.
Sample size/Power	• The stepped-wedge CRCT is considered to have higher power and precision with fewer clusters and increasing number of steps.	• In reality, the power, number of clusters and relative sample size is far more complex	• Achieving sample size and power in the design are not the major issue, however the study is limited to a fixed number of clusters and number of TB diagnosed cases within the NTP that occur during the study period
		• The potential required power depends both on local requirements or existing fixed numbers of centres or patient populations.	

### Planning and development of the pragmatic stepped-wedge CRCT protocol

This protocol is a joint endeavor between local NTP partners including the operational managers of the Ministry of Health TB program in SJL and researchers. Indeed such collaborations at the outset of operational studies, like the stepped-wedge CRCT in SJL, are crucial to their success. During the planning stages, a partnership of NTP programmers with local and international researchers was formed with the common goal of estimating the added benefit of actively evaluating HCs of TB cases for disease compared to the existing DOTS TB strategy of symptomatic individuals self-reporting for evaluation (standard of care). NTP programmers highlighted the importance of having a rapid intervention rollout in order to meet annual programmatic targets and to demonstrate public health action across all health centres in their jurisdiction. Typically in many RCT designs, only half of the included study health centers would be assigned to the intervention arm [8]. Researchers emphasized the need to plan study design aspects such as random assignment, sufficient study sample size in intervention or control arms and supervision of health workers to ensure high quality implementation and sound data management. The stepped-wedge design most aligned with stakeholder needs, including the introduction of randomized crossover times and allowing NTP programmers to oversee a controlled implementation of the intervention across all health centres [26]. This novel design is considered particularly useful in the implementation of public health interventions, given the phased implementation steps. However stepped-wedge designs are relatively new, and many programmatic managers may be unfamiliar with their ability to yield methodologically valid and informative results. Finally, an upfront understanding of the utility of this implementation design is needed so findings of this study would considered by local decision makers hearing of the stepped-wedge CRCT design for the first time.

Researchers can advise on methodological design issues in order to meet the needs of the research question, however in order to understand the operational research aspects of the protocol, gaining a thorough understanding of the local daily operations of the TB program at the health centre level is a key aspect to the design of this protocol. This knowledge included understanding of how contact investigations are undertaken routinely, how the intervention will be applied in local NTP centres, and how monitoring, supervision and data collection could be undertaken feasibly throughout the study period.

### Strengths and challenges of the stepped-wedge design

The stepped-wedge design is particularly useful in this study due to the required widescale roll-out of this public health intervention. In SJL, the systematic stepped-wedge implementation of a provider-initiated ACF program amongst HCs of TB cases provides new evidence, where previously very limited pre-existing effectiveness data are available and few, if any, have been measured within the local public health program in a TB endemic area.

While the staggered intervention initiation times increase the flexibility of the stepped-wedge design in practice, it also requires understanding of the methodological complexities involved. A stepped-wedge allows for a degree of flexibility in its design, such as the number of centres at initiation, and enough time between wedges to provide training and achieve enough study power. However, this design is still subject to biases and threats to sample size and study power. Determining the number of clusters, the number of steps and time-frame for rollout must all be predetermined and require special consideration. If the composition of clusters to be randomized at each crossover time point is unbalanced, this could result in skewed increases or decreases in the measured outcomes. For example, if all high TB rate centres were to cross over at the first time point and all low TB rate centres cross over at the last time point, this could result in a skewed numbers of secondary TB outcomes in HCs observed earlier and for a longer time period of the study. A simple stratification for the randomization of health centres by TB rate at each crossover time point is used in the current protocol to account for TB burden and as a proxy for the corresponding size of clinics to evenly distribute centres across the various wedges [30, 31].

A common challenge for many operational research designed studies is that blinding of the intervention assignment is not possible. In the current protocol, health centres awaiting crossover may have anticipated their likely crossover time based on the number of health centres that have already initiated the intervention. Additionally, the public health intervention is undertaken by health centre staff, who along with patients, know whether or not they have initiated the intervention. In our study, contamination between clusters, though possible, is not a major concern as TB patients and their HCs must attend the health centre of the catchment area to which their primary residence is registered.

The selection of a stepped-wedge design is considered optimal in the context of logistical, feasibility and resource challenges. However, the stepped-wedge design of a population-based implementation does not necessarily require fewer research or programmatic resources to undertake adequately compared to other study designs. In the current protocol, given a study period spanning 20 months, resources were required for monitoring the full implementation of the intervention across all centers for the entire time frame. These include study resources for data quality monitoring, training and supervision of the intervention, and data extraction from health centre charts throughout the full study period. While all operational research studies are subject to these challenges, it is a consideration in the implementation of a stepped-wedge design study where intervention cross over times occurs over a long period. If certain centres or the entire district interrupt the intervention in health centres for unforeseen circumstances, such as personnel strikes, public health outbreaks or other political challenges, then data collection, quality and completeness could suffer and at minimum require adjustment in the study time lines and addition of resources to complete data collection or worse, could lead to imbalances or biases in the overall stepped-wedge designed study.

### Strengths and challenges to the stepped-wedge design during implementation within a routine TB program

There are several advantages and disadvantages to designing a pragmatic stepped-wedge CRCT for operational research within a routine public health program. A unique feature of the current protocol is the use of routine NTP program staff. The stepped-wedge approach using NTP personnel provides an evaluation of intervention effectiveness within actual programmatic conditions and therefore, may be more representatives then a study using strictly dedicated highly trained research personnel. The ability to sustainably integrate the intervention into the program during the study period was facilitated given the intervention was presented as a function of the routine program and not a study specific responsibility. The stepped-wedge implementation provides a useful tool to be able to logistically implement the study, allowing for targeted training within small groups as they initiate the intervention.

Public health interventions are complex and context dependent, as they are integrated within the infrastructure of existing health systems, dependent on local political, socioeconomic and cultural perspectives of the population and its public health practitioners [6]. The use of routine NTP nurses and physicians adds a complexity given that this intervention of household contact tracing is conducted within the context of numerous other responsibilities of the health centre staff. TB programs are impacted by complex treatment management protocols for active TB cases, a high burden of cases in endemic areas and numerous administrative programmatic activities (e.g., reporting forms, indicators, record keeping). Therefore, effective implementation requires planning, high quality training and active monitoring. These factors are better achieved when programs are initiated in a manageable number of sites; the stepped-wedge design allows for flexibility in determining the number of sites initiating a new program at any one time. This aspect is particularly valuable when beginning the new program in all or half of all sites would not be feasible [39]. The randomized allocation of centres to the crossover time point also eliminates preferential assignments due to either health centres’ performance evaluations or other subjective criteria. In the context of the current study, health centre NTP staff appeared motivated and engaged in training and preparation for the intervention once it is known that all centres will have to implement the new program and undergo the same intensive training, monitoring and evaluation processes, instead of some targeted added work assigned only to half of the centres.

Adherence or fidelity to the program and whether the intervention, in its intended form, is systematically applied by staff within a health centre is a distinct challenge particularly in pragmatic trials where routine program staff are undertaking the new intervention as part of their normal duties [6]. From an analytic perspective, intention-to-treat analysis (ITT) is generally the preferred analytic strategy for CRCTs, which considers outcomes based on the random allocation to the intervention arm, regardless of what happened subsequent to assignment. In practice, if there is no reported difference in effectiveness between intervention and control arms, conclusions need to consider whether the ineffectiveness of the intervention is the cause or if possible low adherence of implementers to the intervention is the more likely explanation. To examine this latter possibility, per protocol analyses, which consider actual adherence to the intervention, will also be examined in our study.

Several challenges can occur in undertaking a research study within the daily operations of a public health program, in addition to high staff turnover, worker strikes, outbreaks in other disease areas, and authorization requirements for data access. These substantial issues lead to interruptions of activities, which may not be encountered in studies using dedicated research staff. While planning and design of the stepped-wedge can help to control some of these threats, in some instances these cannot be completely avoided or controlled. The probability of an interruption occurring increase and should be expected when study periods are projected over several months within a routine operational TB program. However, dealing with unforeseen events is a common reality for most public health settings, and the evaluation of the intervention within a pragmatic setting could reflect its likely effectiveness once integrated into practice.

### Concluding remarks

Stepped-wedge designs provide an important option for public health researchers and practitioners to generate intervention effectiveness data that otherwise could remain unmeasured. Typically, stepped-wedge designs are justified when feasibility, logistics and/or limited resources are important practical considerations while still allowing for a randomization process as part of the operational research. Overall, there is no indication that a stepped-wedge design requires fewer resources than other designs; the design requires resources over a longer period of time, yet involves smaller resources for training and monitoring at any given time point during the study. Finally, fidelity or adherence to the intervention may need to be considered during implementation and in the analysis, in order to correctly interpret null or negligible findings of effectiveness.

The current ongoing study will provide invaluable evidence on contextual factors that would not have been possible in traditional study designs. The findings of this study will have implications for the selection of interventions and allocation of resources in TB programming for Peru, and will be a major contribution in the field of TB prevention and TB contact tracing in LMICs.

## Abbreviations

RCT: Randomized Controlled Trial
CRCT: Cluster Randomized Controlled Trial, WHO, World Health Organization
DOTS: Directly Observed Therapy Shortcourses
LMICs: Low and Middle-Income Countries
HIV: Human Immunodeficiency Virus
NTP: National TB Program
HCs: Household Contacts
ACF: Active Case Finding
PCF: Passive Case Finding
ITT: Intention-to-treat
INH: Isoniazid
